# Delimiting MOGAD as a disease entity using translational imaging

**DOI:** 10.3389/fneur.2023.1216477

**Published:** 2023-12-07

**Authors:** Frederike Cosima Oertel, Maria Hastermann, Friedemann Paul

**Affiliations:** ^1^Experimental and Clinical Research Center, Max-Delbrück-Centrum für Molekulare Medizin, Freie Universität Berlin and Humboldt-Universität zu Berlin, Charité – Universitätsmedizin Berlin, Berlin, Germany; ^2^Neuroscience Clinical Research Center, Freie Universität Berlin and Humboldt-Universität zu Berlin, Charité – Universitätsmedizin Berlin, Berlin, Germany; ^3^Department of Neurology, Freie Universität Berlin and Humboldt-Universität zu Berlin, Charité – Universitätsmedizin Berlin, Berlin, Germany

**Keywords:** myelin oligodendrocyte glycoprotein associated disease, imaging, translational research, EAE, animal models

## Abstract

The first formal consensus diagnostic criteria for myelin oligodendrocyte glycoprotein antibody-associated disease (MOGAD) were recently proposed. Yet, the distinction of MOGAD-defining characteristics from characteristics of its important differential diagnoses such as multiple sclerosis (MS) and aquaporin-4 antibody seropositive neuromyelitis optica spectrum disorder (NMOSD) is still obstructed. In preclinical research, MOG antibody-based animal models were used for decades to derive knowledge about MS. In clinical research, people with MOGAD have been combined into cohorts with other diagnoses. Thus, it remains unclear to which extent the generated knowledge is specifically applicable to MOGAD. Translational research can contribute to identifying MOGAD characteristic features by establishing imaging methods and outcome parameters on proven pathophysiological grounds. This article reviews suitable animal models for translational MOGAD research and the current state and prospect of translational imaging in MOGAD.

## 1 Introduction

Myelin oligodendrocyte glycoprotein (MOG) is a minor transmembrane glycoprotein located in the outermost membranes of the myelin sheath (1) that has long been an important target molecule for animal models of demyelinating diseases. Only in recent decades, antibodies against MOG (MOG-IgG) have been identified in people who were previously diagnosed with various other autoimmune-neurological diagnoses such as multiple sclerosis (MS), aquaporin-4-antibody (AQP4-IgG) seronegative neuromyelitis optica spectrum disorder (NMOSD), and acute disseminated encephalomyelitis (ADEM), as well as in isolated and recurrent optic neuritis (ON) and transverse myelitis (TM) (2–6). Furthermore, MOG-IgG can be discovered “false positively” in several other conditions, as demonstrated in several cases of peripheral neuropathy (7, 8) and tumor/lymphoma (9–11). Thus, care needs to be taken as to when MOG-IgG measurement should be performed as well as to the interpretation and consideration of possible differential diagnosis thereof, as has been pointed out in the formal consensus diagnostic criteria for MOG-IgG-associated diseases (MOGAD) that were recently established for the first time (12). Yet, clinical features of MOGAD partly overlap with its differential diagnoses, most importantly NMOSD and MS, delaying the time required until the correct treatment is applied, thus increasing relapse probability. Clinical and imaging studies until now also often included MOGAD patients grouped together with MOG-IgG seronegative patients (for example, as AQP4-IgG seronegative NMOSD), further limiting the discovery of MOGAD-specific features. This is not only true for clinical research: MOG-induced animal models, such as experimental autoimmune encephalomyelitis (EAE), have been widely used as models of demyelinating diseases in general and MS in particular. With the definition of MOGAD as a separate disease entity, it needs to be reevaluated to which extent the generated knowledge from MOG-induced models is specifically applicable to MOGAD versus what should be considered valid for its differential diagnosis (13).

Imaging can significantly aid differential diagnosis early in the disease course and guide the application of cell-based MOG-IgG assays (if available) (14–16). By using a back-translational approach to investigate disease-specific imaging features in preclinical models, imaging can also be used to improve the understanding of (A) distinct pathophysiology by using methods with single-cell resolution and (B) the pathophysiological basis of distinct imaging characteristics by using feature-specific histopathology. This article reviews translational imaging techniques in MOGAD and its animal models. It also discusses the current and potential future relevance of MOGAD-specific animal models and translational imaging for defining distinct pathophysiological features in MOGAD compared with important differential diagnoses, especially MS (17) and AQP4-IgG seropositive NMOSD (18).

## 2 The pathophysiology of MOGAD

There is very little autopsy and/or biopsy material that documents MOGAD pathology specifically (13, 19–22). Furthermore, these studies were conducted mostly on cerebral samples; there is only one case with spinal cord pathology reported (22). Optic nerves are missing in these evaluations. From the presented material, it can be deduced that there are clear histopathological differences discerning MOGAD from both NMOSD and MS, including a CD4+ dominated infiltrate, with fewer B cells, a moderate number of granulocytes (eosinophils and neutrophils), and many/abundant macrophages, some containing early myelin degradation products. While AQP4 and AQP1 were preserved in MOGAD, reactive astrogliosis and even scarring in and around the demyelinating lesions were observed. Axons and oligodendrocytes were unaffected or variably destructed, with a moderate number of axons showing disturbed fast axon transport and axonal spheroids, especially at the lesion rim. Demyelinating lesions occur usually in white matter in a mixed perivenous and confluent pattern of several perivenous lesions, with affection of cortico-medullary junctions and leptomeningeal areas of the cortex as well as the cerebral white matter. Furthermore, there are no “smoldering” radially expanding lesions with microglial/macrophage rim, as would be seen in progressive MS. A meningeal inflammation in 86% of biopsy cases could be seen. The studies, however, do not agree about complement deposition, one describing complement deposition/activation in white matter lesions (13) and the other describing only occasional perivascular-activated complement and IgG deposition (19). In the latter study and the study by Spadaro et al., MOG-dominated myelin loss with preserved oligodendrocytes was observed (20), whereas the previous one did not discern preferential loss of MOG (13). The pathology of one patient with a fulminant MOGAD-like disease including meningoencephalitis and leptomeningeal enhancement and positive MOG-IgG in the cerebrospinal fluid only showed relative axonal sparing, primary confluent demyelination, reactive gliosis, and CD4+ dominated inflammatory infiltrates (22).

There have also been attempts to define the cytokine profile in patients with MOGAD. A study by Nakajima et al. found elevated levels of serum IL-1ra, IL-5, and TGF-α as compared to MOG-negative patients (23). IL-6 was found to be elevated in the CSF of MOG-IgG seropositive children (24). In the study by Bauer et al., serum cytokine levels of MOG-IgG positive/AQP4-IgG positive NMOSD were compared to those measured in MS patients (25). They discovered 36 analytes being increased from MOGAD compared with MS (IL-8, SDF-1a, MCP-1, GRO-a, IL18, MIP-1b, Fractalkine, HGF, IP-10, SCF, VEGF-A, BAFF, IL-7, TWEAK, MIP-3a, M-CSF, CD40L, MMP-1, IL-27, MIG, LIF, MIP-1a, IL-17A, IL-23, TNF-β, IL-1a, IL-6, IL-21, IL-5, MDC, IL-9, FGF-2, Eotaxin-3, IL-10, Eotaxin-2, and IL-31). Only five cytokines differed between AQP4-IgG seropositive NMOSD and MOGAD, all being lower expressed in MOGAD (APRIL, TNFR2, TRAIL, MCP-2, and CD30). No differences were found in MOGAD/NMOSD with regard to disease activity (relapse/remission and amount of relapses), disease course (monophasic/relapsing), treatment modality, sex, or age; however, the availability of clinical data were incomplete.

## 3 Clinical features and clinical imaging in MOGAD

MOGAD affects pediatric and adult patients and shows no sex or ethnic predominance (26). Typical clinical attacks include ON, TM, and, to a lesser extent, cranial neuropathies, brainstem and cerebellar demyelinating attacks, tumefactive brain lesions, mono- and polyfocal CNS deficits, and white matter leukodystrophy-like damage, as well as encephalitis with seizures and neuropsychiatric symptoms (27–30). The most common first manifestation in adults is ON (>55%), whereas the most common first pediatric manifestation is ADEM (with or without ON, >45%) (31–33).

In contrast to the recurrent disease course in MS and NMOSD, MOGAD can be monophasic (~22–56%) (4, 13), preferentially in children (16, 34–36), or recurrent. The current estimation is limited by the short follow-up lengths of published studies, but only one in three MOGAD patients seems to have a relapse within a year after their initial manifestation (3, 4, 37). The risk is higher with steroid tapering and shortly after the initial attack (3, 4, 38, 39). Other longer studies with a small sample size suggest that the long-term risk for recurrent attacks is higher and that attacks can still occur up to >40 years after onset (40, 41). The risk of relapse is lower in pediatric patients; only one in five kids is affected (16, 31, 42, 43). In contrast to MS, clinical progression independent of attacks has not been widely reported in MOGAD so far (41, 44, 45). Histopathological analysis of autopsies/biopsies did not reveal “smoldering” (i.e., slowly expanding) lesions in patients with MOGAD, suggesting a different etiology, if there was a clinically progressive, meaning an attack-independent, disease course in MOGAD as compared to MS. Yet, the current state of research cannot shed light on the possibility of clinical or subclinical progression in MOGAD (46, 47). In a few cases in our outpatient clinic, we observed that patients experience relapse-free worsening of their symptoms over time; however, a thorough investigation on this matter is still needed.

### 3.1 Brain and brainstem

Cerebral manifestations and imaging findings in MOGAD are diverse. In adults with MOGAD, brain MRI findings are usually sparse and rarely occur in isolation without cerebral syndrome or concurrent optico-spinal lesions (3, 48). Silent lesions are seen in <5% of adult MOGAD patients and even those are usually associated with subsequent relapses (49). Cortical and infratentorial lesion locations are the most common, but large T2-hyperintense white matter lesions can occur (13, 48). In rare cases, tumefactive lesions with a risk for herniation are seen (50).

People with MOGAD have a higher frequency of cortical and juxtacortical lesions compared with people with AQP4-IgG seropositive NMOSD. Yet, the number of lesions in MOGAD is usually lower than in MS, especially at onset (3). Matthews, Juryńczyk and colleagues specifically proposed that lesions close to the lateral ventricle and/or in the inferior lobe, subcortical U-fiber lesions, and Dawson's finger-type lesions strongly suggest a diagnosis of MS vs. MOGAD (51, 52). For infratentorial lesions, the brainstem, especially the pons, close to the 4th ventricle and the middle cerebellar peduncle, are the most common locations in MOGAD — lesions can be found in up to 30% of patients (3, 48, 53, 54). Lesion demarcation is usually poor and lesions can disperse over time (4, 48). Particularly, lesions in the middle cerebellar peduncle can distinguish MOGAD from MS and AQP4-IgG seropositive NMOSD (54). Area postrema syndrome, however, is less common in MOGAD compared with AQP4-IgG seropositive NMOSD (55–57). In contrast to MS and AQP4-IgG seropositive NMOSD, the application of gadolinium rarely reveals a lesion enhancement pattern in MOGAD but can lead to unspecific leptomeningeal enhancement around the brainstem or in uni- or bilateral cortical areas, especially in MOGAD with cortical encephalitis.

In pediatric patients, the most common onset syndrome is ADEM, which typically presents on MRI with large asymmetric and diffuse, supra- and infratentorial T2-hyperintense white matter lesions (58–60). ADEM can also rarely occur in adults—with similar MRI features. Compared with MOG-IgG seronegative ADEM, MOGAD-ADEM more often involves the thalamus (61). MOG-IgG-associated autoimmune encephalitis, a second common pediatric manifestation, presents with large subcortical and/or cortical lesions (31, 62). In contrast to autoimmune encephalitis with other antibodies, normal MRI findings in MOG-IgG-associated autoimmune encephalitis are rare (63). A leukodystrophy-like phenotype of MOGAD, a rarer pediatric manifestation, also presents with large symmetric confluent white matter lesions, yet they are usually clinically progressive (47).

Advanced MRI techniques have been used for a limited number of MOGAD studies so far. Combining fluid-attenuated inversion recovery sequences (FLAIR) with traditional MRI metrics, hyperintense cortical lesions and numerous T2-hyperintense lesions in various locations were identified, respectively, in a subgroup of MOGAD referred to as FLAMES (*FLAIR-hyperintense lesions in anti-MOG-associated encephalitis with seizures*) (29, 64, 65). FLAMES can further be characterized by hyperperfusion of lesions on single photon emission computed tomography (SPECT) (56). Using diffusion-tensor imaging (DTI) and resting state functional MRI, reduced axial diffusivity in line with microstructural white matter damage, and interhemispheric functional connectivity changes of the motor, sensorimotoric and frontal lobe networks, respectively, were identified in MOGAD compared with healthy controls (66, 67). Applying volumetric analyses, no loss of gray or white matter was observed in adult MOGAD patients compared with healthy controls (66, 68). In pediatric ADEM, however, the brain volume as well as the expected brain growth were reduced (69). So far, no advanced MRI marker has been suggested to distinguish MOGAD from its differential diagnoses.

### 3.2 Spinal cord

TM in MOGAD can manifest as sensory, motor, and sphincter dysfunctions (70). It can occur in isolation or combined with other manifestations such as ADEM or ON. Despite often severe impairment in the acute stage, most patients have a good recovery. Yet, especially sexual, bladder, and bowel dysfunction can remain (44, 71). Persisting pain or spasms are uncommon and seen more often in AQP4-IgG seropositive NMOSD than in MOGAD. The MOGAD-associated spinal cord involvement in adult and pediatric patients is largely comparable (72).

**Figure 1:**
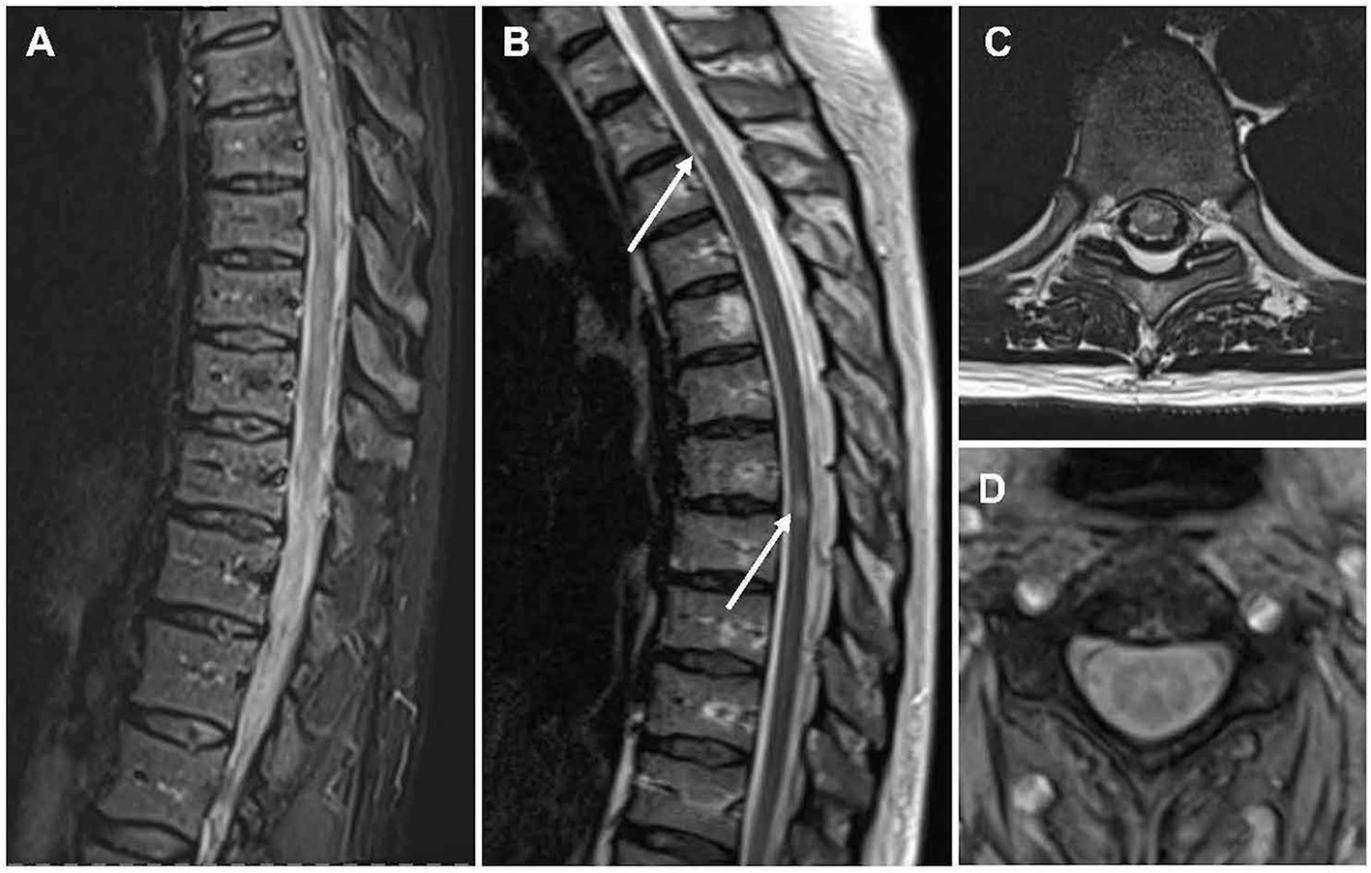
MR imaging of the spinal cord in people with MOGAD. Sagittal T2-weighted MRI showing hyperintense lesions in line with **(A)** an LETM and **(B)** shorter lesions. Axial T2-weighted MRI showing **(C)** a centrally located lesion and **(D)** the characteristic H-sign. LETM, longitudinally extensive transverse myelitis; MOGAD, myelin oligodendrocyte glycoprotein antibody associated disease; MRI, magnetic resonance imaging.

Initial spinal cord MRI can be normal in 10% (73, 74). The most common finding on spinal cord MRI in MOGAD, however, is the so-called longitudinally extensive transverse myelitis (LETM) presenting as a hyperintense T2-lesion spanning over three or more segments and mainly affecting the cervical and/or thoracic cord (Figures 1A–D) (74–77). LETMs rarely occur in MS (78). While LETMs can also be seen in AQP4-IgG seropositive NMOSD, MOGAD patients present more often with multiple lesions and conus involvement (75, 79–81). Also, shorter TM, as typical for MS, can be seen in MOGAD and is more common compared with AQP4-IgG seropositive NMOSD (77, 79, 82).

Up to 75% of lesions in MOGAD are centrally located and up to 50% of lesions are restricted to gray matter, which can often be identified as the characteristic H-sign on axial scans (Figures 1C, D) (73, 79, 81). This is particularly interesting since MOGAD is a highly inflammatory condition primed to the white matter. As discussed below, data from rodent models suggest that this severe white matter inflammation correlates with gray matter hypoxia and increased variation in oxygenation in the gray matter potentially leads to gray matter damage (83), as has been similarly suggested in MS (84, 85). Still the pathomechanism of gray matter damage remains to be elucidated and more autopsy/biopsy samples, especially in MOGAD, need to be analyzed to this end. In contrast to both MS and AQP4-IgG seropositive NMOSD, gadolinium-enhancement is less common in MOGAD (~50%) (72, 75). However, contrast enhancement of the pia and cauda as well as contrast enhancement and thickening of dorsal nerve roots can occur (28, 72).

The application of advanced spinal cord imaging in MOGAD has so far been very limited. Spinal cord atrophy as measured by volumetric MRI has been only seen after severe attacks (86, 87). Silent spinal cord lesions can occur during an attack of the brain or optic nerve but are extremely rare outside of attacks in MOGAD, making spinal cord involvement outside of acute attacks unlikely (49).

### 3.3 Retina and optic nerve

Optic neuritis (ON) is the most frequent onset feature in adults and one of the most common manifestations of MOGAD in general (88). Thus, imaging of the visual system is a promising approach for diagnosis and differential diagnosis (89, 90). In MOGAD, ON is often bilateral and mostly located in the anterior segment causing severe edema (39, 91, 92). Although single ON attacks often do not lead to tremendous retinal neurodegeneration, the high frequency of attacks in MOGAD can accumulate significant damage (92). Due to its severe symptoms, silent ON is uncommon in MOGAD, yet a bilateral ON can remain unrecognized due to stronger symptoms in one eye.

**Figure 2:**
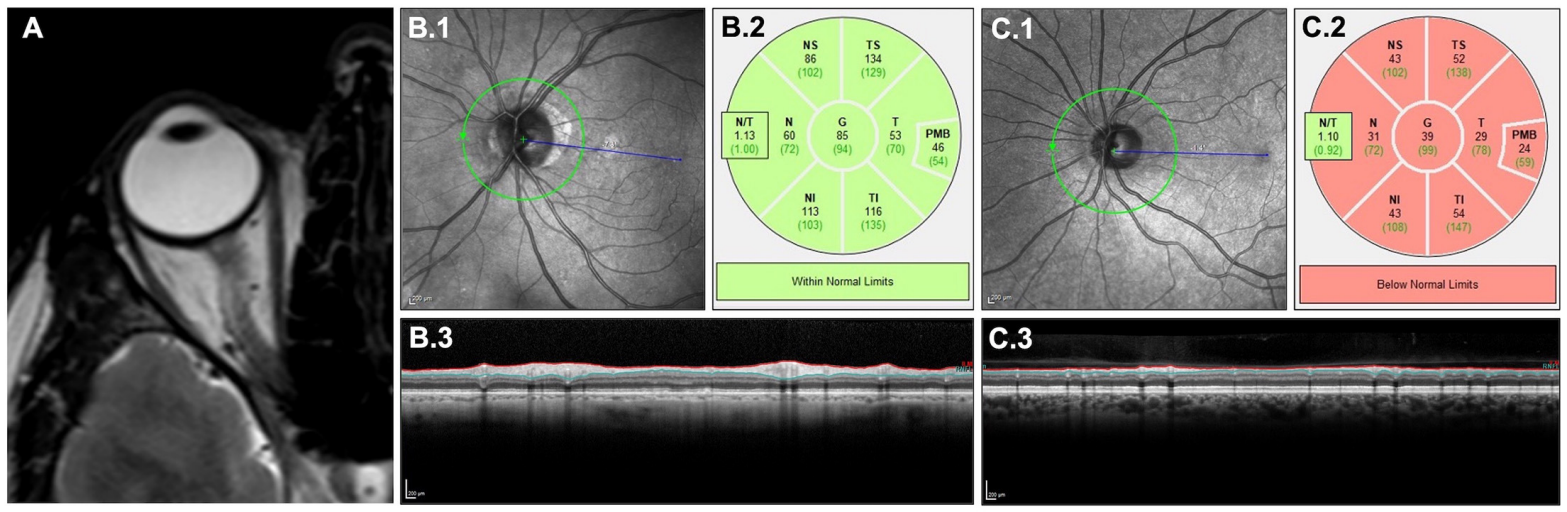
Clinical imaging of retina and optic nerve. T2-weighted MRI of the optic nerve **(A)** showing a longitudinal lesion with edema. OCT quantifying retinal neuroaxonal content measured by pRNFL around the optic nerve head in a retina without a history of ON **(B)** and with a history of ON **(C)** in MOGAD: scanning laser ophthalmoscopy **(B.1, C.1)**, color-coded comparison with a healthy control cohort **(B.2, C.2)** and cross-sectional B-scans showing pRNFL atrophy in **(C.3)** compared with **(B.3)**. MOGAD, myelin oligodendrocyte glycoprotein antibody associated disease; MRI, magnetic resonance imaging; OCT, optical coherence tomography; ON, optic neuritis; pRNFL, peripapillary retinal nerve fiber layer.

Lesions on optic nerve MRI usually show T2-hyperintensity and gadolinium enhancement on T1-weighted imaging (Figure 2A). In MOGAD, drastic nerve swelling and characteristic perineural/periorbital gadolinium enhancement are often seen (93, 94). Hemorrhages can occasionally occur, particularly in peripapillary regions. Optic nerve lesions are also extensive, involving more than half of the pre-chiasmic optic nerve, which distinguishes optic nerve lesions in MOGAD from shorter lesions in MS (95, 96). The optic nerve MRI can show the characteristic anterior involvement, which distinguishes optic nerve lesions in MOGAD from the also often extensive but mostly posterior lesions in AQP4-IgG seropositive NMOSD (96, 97). Simultaneous bilateral involvement is more common in MOGAD than in both MS and AQP4-IgG seropositive NMOSD (98).

ON leads to retrograde retinal neurodegeneration, which can be monitored using spectral domain optical coherence tomography (OCT). OCT is a non-invasive imaging method using the interference of low coherent light to produce high-resolution images of the retina (99). Neurodegeneration after ON is quantified by OCT measuring the peripapillary retinal nerve fiber layer (pRNFL) and the combined ganglion cell and inner plexiform layer (GCIPL), which contain the axons and cell bodies of retinal ganglion cells, respectively (Figures 2B, C) (99, 100). Whereas, the pRNFL usually undergoes swelling during the acute phase before experiencing volume loss due to subsiding edema and concurrent degeneration; the GCIPL is less affected by swelling and undergoes a steadier volume loss due to neurodegeneration. According to the current consensus, the majority of retinal neurodegeneration happens within the first 6 months after the acute ON attack independent of the underlying disease. Yet, the acute pRNFL swelling in MOGAD is described to be more severe and is suggested as a diagnostic marker distinguishing MOGAD from MS (101). This might lead to a prolonged (more than 6 months) pRNFL reduction in MOGAD (102, 103).

To diagnose a history of ON, the use of the absolute or relative differences in pRNFL and GCIPL between both eyes of patients, the so-called inter-eye-difference (IED), has been suggested (104). Due to a higher frequency of unilateral ON, the diagnostic value of IED is very high in MS and reasonable in NMOSD (105–108). Yet, the use of IED has not been investigated in MOGAD and seems limited due to the high frequency of bilateral ON. When comparing absolute values of pRNFL and GCIPL after ON, MOGAD patients usually have more severe retinal neurodegeneration (ergo thinner pRNFL and GCIPL) than patients with MS. pRNFL and GCIPL after ON are comparable in people with MOGAD and AQP4-IgG seropositive NMOSD (109). Yet, several publications suggest that the neuronal loss per ON is lower in MOGAD, and only the higher frequency of ONs leads to damage that is comparable with AQP4-IgG seropositive NMOSD patients with less frequent but more severe ONs (109, 110). Despite the neuroaxonal loss being comparable, people with MOGAD often have a better long-term visual outcome compared with AQP4-IgG seropositive patients — the pathophysiological explanation for this difference is still pending (111–115).

Retinal and optic nerve damage independent of ON has been shown in MS, where it can also be used to predict disease activity (116–120) and, to a lesser extent, in AQP4-IgG seropositive NMOSD (121–126). Advanced OCT imaging suggests that ON-independent retinal changes in AQP4-IgG seropositive NMOSD are related to primary astrocytopathy (127). So far, no ON-independent neurodegeneration above aging-related standard and no primary and/or outer retinopathy has been shown in MOGAD, potentially aiding differential diagnosis (102, 103, 128). First applications of OCT angiography showed a significant decrease in vessel density after ON in MOGAD, which exceeded the changes in AQP4-IgG seropositive NMOSD (129, 130). A new generation of advanced OCT imaging methods including 3D-shape analyses and feature recognition can potentially contribute to a better understanding of ON-dependent and -independent changes in MOGAD and their use for differential diagnoses in the future (131–134).

## 4 Are MOG and MOG-IgG-induced animal models good models for MOGAD?

Animal models that induce encephalitis to mimic autoimmune-mediated disease in the CNS include approaches of active immunization, passive transfer, antibody (co-)mediated disease induction or exacerbation as well as transgenic/genetic modifications to mention the most common ones. MOG-mediated disease is one of the most commonly used to model MS and has been used in many variations that have been described and reviewed extensively elsewhere (135–164). However, with the emergence of MOGAD as a separate disease entity and considering that these models do present drawbacks in reproducing MS characteristics (mainly CD4+ mediated, no MOG-IgG present in any form of MS, etc.) (2, 12, 35, 165–168) the issues in their translation into new therapeutic modalities for MS could be viewed in a new light (169–171). In this chapter, we will discuss to what extent (some selected) MOG-induced animal models as well as some non-MOG-induced models resemble human MOGAD disease and to what extent they could be employed for diagnostic, prognostic, and therapeutic approaches (135).

### 4.1 MOG-induced animal models

The course and development of EAE are dependent on many different factors and their ratio to each other (172) including the conformation, concentration, solubility, specificity of the antigen used (135), age, species and genetic background of the experimental animals (139, 173–175), the adjuvant (176–179), and timing of immunizations/transfer to name just some variable instances. It has been shown, for instance, that the disease course—monophasic, relapsing, primary/secondary progressive, or chronic progressive (with disability accrual)—can be regulated by the immunization protocol of Lewis (LEW.1AV1) rats with MOG (135).

### 4.2 MOG-IgG-mediated models

In patients, MOG-IgG was shown to be present during very early stages of disease onset and to persist over long periods of time even during remission. The MOG-IgG titer is dependent on disease activity; however, the antibodies cannot independently induce the disease. In contrast, MOG-IgG has been found in early, intermediate, and late stages of EAE; however, the titer was not disease activity-dependent, being low at the beginning and higher in the end, with the amount being similar during the acute and remission phases (135). Complement-mediated pathology/demyelination could be induced in EAE (180, 181) in line with findings of complement deposition in MOGAD autopsy material. In a constitutively MOG-IgG-producing transgenic mouse model, EAE could be induced in the absence of B cells but required T cells (182).

Experimental studies suggest that MOG-IgG mediates a pathogenic effect in EAE (181, 183, 184). It seems, however, that circulating MOG-IgG require the presence of complement, cytokines, and/or a (T cell-induced) inflammatory milieu to trigger demyelination/enhance inflammation via CDC/antibody mediated cellular cytotoxicity (ADCC), as alone, they are not able to do so (152, 185–187). It was shown in naive recipient animals that primary demyelination restricted to CNS nerve fibers could be induced via injection of a monoclonal MOG-IgG (the 8-18C5) into their cerebrospinal fluid. In adult Sprague-Dawley rats, an association between antibody titer and degree of demyelination could be demonstrated after infusion of sera from Hartley guinea pigs previously immunized with homologous spinal cord lysate in adjuvant into their subarachnoid space. The presence of MOG-IgG in injected sera was demonstrated via an anti-MOG ELISA (185). The direct translational value of these experiments seems tenuous as the blood-brain barrier (BBB) was circumvented in these experiments. The demyelinating effect of antibodies directed against MOG was also demonstrated in a Sprague-Dawley animal model in which monoclonal MOG-IgG-producing B cell hybridomas were implanted into the right lateral ventricle (188). MOG-IgG titers could not be linked to disease outcome in MOGAD patients to this date (189); however, a longitudinally persistent MOG-IgG positivity seems to be associated with a higher risk for relapse (16, 34, 42, 190, 191). Furthermore, it was shown that children with monophasic ADEM lose MOG-IgG over time (192). This is mirrored in animals as high frequencies of relapses are associated with permanent damage. MOG-IgG injection was lethal when injected into SJL mice repeatedly challenged with passive MBP-specific T cell transfers (mimicking a relapsing disease course) that had not yet completely recovered from the previous relapse as opposed to no negative effect of the antibody if the disease score was zero (193). In another experiment with repeated passive transfer of T cells and subsequent antibody application, formation of large demyelinating lesions accompanied by lack of remyelination could be observed, with pronounced astrocytic scar formation traversed by “naked” axons, both characteristic of MS, and not described thus in the available MOGAD autopsy/biopsy cases (194). In mice engineered to produce high MOG-IgG titers, pathology could only be seen after immunization with MOG antigen, without regard to the genetically more (SJL) or less (C57BL/6) EAE-susceptible background (195). This is in line with experiments showing that B cells are not critical for the development of MOG-induced EAE (B cell-deficient muMT mice on C57BL/10 and DBA/1 genetic backgrounds and X-linked immunodeficiency (xid) mice on DBA/1 background) but contribute to the severity, i.e., demyelination rather than inflammation (196). However, the effect of the autoantibodies seems to differ regarding their enhancing characteristics of demyelination/inflammation depending on the agent EAE was induced with. Thus, MOG-specific T-cell-mediated inflammation can be enhanced via augmented antigen presentation (197), whereas in EAE induced by non-MOG-specific T-cells, demyelination is triggered but no enhancement of inflammation is observed (184).

### 4.3 Animal models targeting MBP

In the passive transfer EAE model (transfer of antigen-specific T cells propagated *in vitro*) with intravenous injection of MBP-specific T cells and subsequent intravenous (i.v.) MOG-IgG injection at the onset of the disease, a massive augmentation of clinical affection as well as primary demyelination could be observed in Lewis rats. Similarities to MOGAD include lesions located predominantly in the spinal cord and medulla oblongata at circumventricular organs (BBB is more transmissible at these points), predominantly mononuclear cell infiltrate with some granulocytes, perivascular, or focal confluent demyelinated lesion formation (75% of T cells infiltrate to the parenchyma), depending largely on the amount of injected T cells, extensive gliosis, preservation of axons, and remyelination of demyelinated lesions (20, 151, 198). There is a clear macrophage-dominated infiltrate seen in MBP EAE (macrophage: T cell ratio of approximately 6:1); in some cases of MOGAD histopathology, the amount of both cell types seems to be near to equal (1:1.2, respectively) (19), while in others, T cells seem to be somewhat outnumbered by macrophages, especially in the parenchyma [no ratios given, (13, 22)]. Furthermore, the relevance of complement involvement, in the form of membrane attack complex (MAC) formation as well as ADCC, was demonstrated in this model as well as MAC formation in PVG/c rats (±C6 complement component, immunized with guinea pig myelin basic protein (gpMBP) and Complete Freund's Adjuvants (CFA) containing *Mycobacterium tuberculosis* H37Ra) (199), which is in line with findings of complement deposition, to varying degrees, in MOGAD patients‘ biopsies/autopsies (200, 201).

Active EAE to MBP immunization has been induced in Lewis rats with subsequent MOG-IgG injection (MoAb 8-18C5) 10 days after sensitization. Antibody injection led to significant worsening of clinical and histopathological observations compared to the disease course without the addition of antibody (193, 202), granulocytic infiltrate, perivascular complement deposition, and inflammatory cuff formation, which could be observed similarly to histopathology found in MOGAD patients. The disease course after MBP immunization, with or without subsequent antibody injection, was monophasic; progression or relapse was not recorded after an observation period of 13 weeks (193).

### 4.4 Animal models induced by MOG-specific T cells

In animals (Lewis rat) with passive MOG-EAE (T cells raised against the MOG_35−55_ peptide, with and without MOG-IgG transfer), inflammatory changes were induced in the spinal cord without producing an according clinical correlate of typical EAE symptoms (tail tonus loss, gait instability, and severe weight loss) (173, 203). The macrophage: T cell ratio was clearly shifted toward T cells (1:6, respectively), and a few cells (7–20%) of the inflammatory perivascular infiltrate left the perivascular space toward parenchymal infiltration. Contrary to all previously analyzed passive EAE models [induced with MBP, S100β, PLP nicely reviewed in (140, 204, 205)], no peripheral affection was noted. Severe blood-brain barrier dysfunction was induced by passive MOG-EAE and subsequent intravenous injection of a demyelinating MOG-specific monoclonal antibody that induced severe clinical disease. Furthermore, it has been shown that the location of lesions was dependent on the antigen used to raise the T cells (204).

### 4.5 Animal models induced by MOG peptide immunization

Immunization (active MOG-EAE) in Lewis rats via a highly purified recombinant protein, mMOG, spanning its N-terminal domain (a.a 1–125 + CFA) failed to activate immunodominant T cell epitopes, producing an inflammatory non-demyelinating phenotype as seen previously with passive transfer EAE (206). No clinical symptoms could be observed, at least partly attributed to reduced macrophage recruitment as compared to immunization with MBP/PLP protein/peptide (173). Antibodies to MOG_1−25_ were induced by mMOG immunization and production could be enhanced by repeated immunization (booster) after 4 weeks; however, this epitope does not seem to produce a demyelinating phenotype. Again, extensive perivascular and subpial demyelination could be produced by co-injection of the MOG-specific mAb 8-18C5 on day 10 post-immunization. Thus, immunization with mMOG seems to reproduce MOGAD histopathology rather poorly. Contrary to these findings, immunization with MOG isolated from human/rat brain tissue as well as immunization with MOG_35−55_ peptide were able to induce a severe relapsing-remitting disease course in Lewis rats presenting with inflammatory demyelinating lesions and perivascular cuffs (mononuclear, including myelin debris) with accompanying MOG specific IgG production (in the former) (207). Different rat strains (BN, DA, Lewis.1N, Lew1AV1, and Lew1A) were challenged with different MOG compositions [soluble or precipitated in complete or incomplete Freund's Adjuvants (CFA/IFA)] and varying immunization protocols (205). This study shows a very good reproduction of core MOGAD characteristics, more or less expressed depending on strain/regime/immunogen composition, in all the animals. These include the development of a chronic relapsing disease course in 111/156 animals, 16/156 developed chronic progressive disease, 17/156 showed stable course with neurological deficit. Development of predominant or selective ON was seen in some animals. Neuropathology (in 133/156 animals) featuring perivenous inflammation, confluent demyelinating plaques with complement deposition at sites of active demyelination, relative axonal sparing, inflammatory perivenous infiltrates, and meninges with parenchymal infiltration adjacent to the pia mater with predominant T cell/macrophage infiltration as well as polymorphonuclear infiltrates (mostly in animals with ON/spinal cord affection) and frequent remyelination. Of the observed pathology, glial scar formation is not readily found in current reports of MOGAD histopathology. Acute disseminated leukoencephalomyelitis was seen in the other 23 animals, which is about ~15 of animals; in comparison, in human children ADEM occurs in >45% cases and in adults in ~10%, featuring severe perivenous inflammation and little/absent demyelination. Major patterns of lesion distribution across the CNS (optic nerve/spinal cord, isolated ON, spinal type, cerebellar type, periventricular type, acute disseminated leukoencephalomyelitis type, and destructive transverse myelitis) go along well with lesion distribution seen in MOGAD (classified by these authors at that time as neuromyelitis optica). In this study, the authors showed that optic nerve involvement was independent of MHC genes; in addition, it was shown by others that MHC haplotype seems to influence disease susceptibility to a certain amount (174, 208). Differences in these models compared to MOGAD were seen in relation to sex-associated characteristics, specifically in DA rats. It could be observed that female rats had a high incidence of ON, whereas none was seen in male rats. In a study by the Mayo Clinic, the authors observed that of the 87 MOGAD patients presenting with ON, 57% were female (92). Another clear sex difference was seen in eosinophilic granulocyte infiltration, which was seen only in female rats; however, this phenomenon was not mentioned by any of the MOGAD autopsy/biopsy studies cited above. In a transgenic mouse study with MHC II-restricted animals, immunodominant MOG epitopes were identified and EAE could be induced (209). This is in line with findings that CD4+ T cells (HLA class II) dominated cell infiltrates in MOGAD patients' lesions. In a Dutch and UK study, no negative association of MOGAD to an HLA subtype could be discerned to date, whereas a Chinese study suggested an association of DQB1^*^05:02-DRB1^*^16:02 alleles to pediatric-onset MOGAD (210–212). Notably, to this date, no definite genetic association could be shown in MOGAD; specifically, no strong HLA dependence, which is in contrast to what has been suggested in MS (210–212).

One of the most widely used EAE animal models to date is the C57BL/6 mouse MOG_35−55_ EAE (213, 214). Similar to MOG-induced EAE in Lewis rats, injection with only MOG_35−55_ peptide (and CFA, with and without *pertussis toxin* PT) was able to induce neurological impairment in C57BL/6J mice featuring a chronic, non-remitting disease course and mild clinical presentation usually restricted to paralysis in the tail and hind legs. Mice did not recover after immunization even after long-term observation (3 months), which, however, could not be observed in other studies (215). Lesions included perivascular infiltration of mononuclear cells and secondary demyelination. PT was observed to enhance EAE moderately and lead to earlier disease onset, however, PT is not needed to induce overt clinical disease *per se* (213).

It was shown in active C57BL/6 mouse MOG_35−55_ EAE (with CFA and PT) that natural killer cells (NK-cells) are involved in preventing EAE development, as Th1 response (including Th1 specific cytokine production, IFN-γ, and TNF-α) seemed to be elevated in NK-cell depleted animals (216). However, a reduced amount of NK-cells could only be seen in NMOSD but not MOGAD when compared to each other (217).

There have been some attempts to define the cytokine profile in patients with MOGAD (see above). In a study using actively induced MOG_35−55_-EAE in mice (induced with MOG_35−55_, CFA, PT) changes in cytokine production largely overlapping with MOGAD (IL-4, IL-6, IL-10, IL-12, IL-17, IL-23, TNF-α, IFN-γ and TGF-β) were observed (218). In EAE, the involvement of IL-6 has been extensively studied. It has been shown that IL-6 (conditionally) deficient mice are resistant to EAE (219–221), that IL-6 is involved in the induction phase of EAE (222) (MOG_35−55_ induced), that IL-6 inhibits T cell conversion to the Treg phenotype (Foxp3+) (223), and is (224, 225) or is not (223) involved in conversion to Th17 type T cells. It has been shown that tissue damage occurs preferentially at sites of IL-6 production (226, 227) and that induced antibodies against IL-6 are protective against EAE (228). Interestingly, PT which is often used to enhance EAE has been shown to induce IL-6 (229). In a mouse line deficient in the IL-6 gene (129/SvXC57BL/6), immunization with MOG_35−55_ peptide showed abrogated EAE induction (230). These findings are in line with the seemingly beneficial effect of Tocilizumab/Satralizumab (recombinant, monoclonal antil-IL6 receptor antibodies) on relapse prevention in MOGAD patients (231–236). IL-23 involvement was shown in EAE induction (237), as well as the development of Th1 and Th17 cells (238–240) but is not necessary in the effector phase of the disease.

It was demonstrated in different rodent animal models that IL-10 is involved in EAE via increased disease severity when deleted, and IL-10 contributed to disease course duration (shorter) and recovery (241, 242). In a passive transfer EAE with anti-MOG T cells into MyD88 animals, it was shown that resistance to EAE was mediated via the secretion of IL-10 by recipient T cells (243). Further, it was shown that immunization with MOG_35−55_ in susceptible (SJL and NOD) vs. resistant strains (B10.S or III) differed in the amount of cytokines produced, resistant strains secreting primarily IL-4/IL-10 and transforming growth factor (TGF)-β, vs. susceptible strains with predominant IFN-γ production (244). In contrast, 129/Sv mice knocked out for the gene coding for the ligand-binding chain of the IFN-γ receptor developed severe EAE (129/Sv are resistant to MOG-induced EAE), indicating that IFN-γ was involved in ameliorating EAE during both the effector and induction phase (245, 246). IFN-γ involvement in the determination of lesion location was shown in passive MOG-EAE induced in C57BL/6 mice lacking the IFN-γ receptor (IFNγR) (247) and it was shown that CFA/PT alone do not induce IFN-γ production, but immunization together with MOG is necessary (248).

The above-described patient cytokine profile points toward the direction of TH17 involvement (IL-17A, IL-23, IL-6, and IL-21) in the pathogenesis/disease course of MOGAD (249). The involvement of Th17 T cell subsets has been under discussion since their discovery in 2005 (250, 251), allocating a role for them in EAE induction/autoimmunity (252–256) or not (257) in different MOG-induced animal models [reviewed elsewhere (249)], going so far as to implicating the intestinal microbiome to EAE resistance of mice deficient in IL-17A and IL-17F (258). In a passive transfer model with MOG-specific T cells derived from 2D2 mice, it was shown that both Th1/Th17 cells are able to induce EAE; however, Th17 induce an atypical phenotype in half the cases (beginning with ataxia instead of paralysis, only developing paralysis later). Interestingly, histopathology [severe immune cells infiltration (CD4+ T cells and macrophages), astrogliosis, microglia activation, demyelination, and axonal damage] as well as lesion location (throughout the CNS as well as inflammatory infiltrates/demyelination in the PNS) were similar in both Th1 and Th17 recipients (259). A higher frequency of ataxia was found in children with ADEM positive for MOG-IgG compared to MOG-IgG negative cases (60). No involvement of IL-5 could be detected in the initiation or effector phases after immunization of C57BL/6J (or IL5^−/−^) mice with MOG_35−55_ (260). Likewise, IL-21 was found irrelevant for Th17 induction (261).

Cerebral cortical encephalitis is one of the core clinical demyelinating events suggested by Banwell et al. in the diagnostic criteria for MOGAD (12). Current models of EAE do not reflect cortical demyelination ideally. One model trying to recapitulate these lesions targeted the cerebral cortex by stereotactical injection of pro-inflammatory mediators into Lewis rats challenged with MOG_1−125_ (262). Inflammatory, demyelinating lesions were induced including complement deposition, and as seen in MOGAD autopsy cases, ready remyelination was observed. In a model of Dark Agouti rats immunized with MOG, inflammatory agents were injected into the subarachnoidal space to avoid parenchymal damage. Here as well, IgG and complement deposition were observed, the amount of inflammatory infiltrate was little and mostly limited to meninges, and as in the model described by Merkler et al., repair was rapid (263).

### 4.6 Transgenic animal models

There is a wealth of genetically modified/transgenic/humanized animal models that have been reviewed in more detail elsewhere (264–266). We will discuss some of those models in this review in regard to their similarities as models for MOGAD. In MOG_35−55_-induced active EAE in non-obese diabetic (NOD) mice, some groups showed that a switch from relapsing-remitting (RRMS) to secondary progressive (SPMS) can be induced and this model is considered to reflect the pathology of SPMS well (267, 268). Other groups could not observe the switch of clinical symptoms to a progressive disease course (269). When disease was induced in NOD mice via immunization with MOG_35−55_ and CFA/PT, inflammatory/demyelinating lesions developed preferentially in brain white matter (fimbria/internal capsule) and also in the spinal cord with macrophage infiltration. Microglia/astrocyte activation could be observed (268). Interestingly, disease development and progression could be prevented via anti-IL-12 antibodies in this model (270). NOD mice with transgenic TCR recognizing MOG_35−55_ were generated (1C6 TCR) (267) and showed development of spontaneous optic neuritis/EAE in around 1% of the animals, distributed similarly in both male and female animals. Upon passive transfer EAE, these mice developed preferentially spinal cord lesions and optic neuritis. When immunized with MOG_35−55_ and CFA, these mice developed chronic disease after the second relapse with CD4+ T cells predominating over CD8+ T cells at a ratio of 30:1 in the lesions, with elevated production of IFN-γ and IL-17. In following experiments, 1C6 TCR mice were crossed with Ig heavy-chain knock-in mice (IgH^MOG^ or Th mice) on a C57BL/6 background (195). IgH^MOG^ mice harbor autoreactive B cells producing anti-MOG antibodies with the heavy chain of the 8.18C5 demyelinating MOG-specific antibody; however, they do not develop spontaneous disease but were shown to both accelerate and exacerbate EAE irrespective of the inducing agent. The frequency of spontaneous disease was higher in 1C6 x IgH^MOG^ mice (45% males, 79% females), CD4+ T cells still outnumbering CD8+ T cells 7:1, the amount of CD8+ T cells, however, being higher compared to 1C6 TCR mice. Lesions were located mostly in the spinal cord, with around 40% of the mice showing optic nerve lesions, and no formation of ectopic follicle-like structures was observed in the CNS of the animals. A large part (75%) of asymptomatic 1C6 × IgH^MOG^ animals showed exclusively cerebellar lesions upon histopathological examination.

Also, in the Biozzi EAE model (271), chronic relapsing disease could be induced via subcutaneous injection at days 0 and 7 in both hind flanks with an emulsion spinal cord homogenate and CFA complemented with *M. Butyricum*. In these animals partial closing of the BBB, meningeal ectopic lymphoid tissue with adjacent subpial demyelinating lesions and a switch from T cell, to B cell, predominance and serum MOG-IgG generation in later chronic disease stages could be observed (272).

Another study in a transgenic mouse model, GFAPγR1Δ, induced EAE by active immunization with MOG_35−55_ to gain a progressive phenotype with sustained inflammation and increasing clinical disease. This study suggests that tumor necrosis factor (TNF) is predominantly produced by CNS infiltrating macrophages rather than microglia after the acute disease stage (273). Contrary to promising preclinical results of TNF blockade, however, the success of TNF suppression in MS patients did not yield uniformly positive results (274). For MOGAD in relation to TNF treatment, little is known and data from a small retrospective study (*n* = 5) is inconclusive regarding negative effects, however also no clear positive outcome is documented (275). Primary progressive-EAE (PP-EAE) was further established in A.SW mice sensitized with MOG_92−106_ and SJL/J mice sensitized with MOG_92−106_ and curdlan (276). A.SW mice develop large areas of demyelination, immunoglobulin deposition, and neutrophil infiltration in the absence of a T cell infiltrate (14, 16) while SJL mice show T cell infiltration and paralysis. Both models generated an anti-MOG antibody response (276).

Another model is the “genetic 2D2” EAE model (TCR^MOG^) in which mice were generated with a TCR that is directed against MOG_35−55_ (with a C57BL/6 background), about 5% of the animals develop EAE spontaneously with inflammatory/demyelinating lesions in brain, spinal cord, and optic nerves (277). Furthermore, a large proportion of non-clinically symptomatic mice showed ocular abnormalities, and around 15% of the 2D2 transgenic mice developed isolated optic neuritis in the absence of clinical/histological signs of EAE. These lesions showed macrophage infiltration, demyelination, and axonal damage. Interestingly, the challenge with PT alone was sufficient to induce clinical EAE in 39% and histological EAE in 56% of 2D2 mice. The GF-IL23 model, with astrocyte-specific IL-23 secretion on a 2D2 background (most CD4+ have TCR specific for MOG_35−55_), showed a spontaneous EAE induction with chronic disease course, clinical affection (ataxia/paraparesis), and a high proportion of B cells. A pronounced B cell accumulation and B cell follicle-like infiltrates have not been reported as such in MOGAD yet (160).

To generate double transgenic opticospinal EAE (OSE) mice (277–280), 2D2 mice were then crossed with IgH^MOG^ (with a transgenic B cell receptor to MOG, described above). The offspring of these mice spontaneously develop ON and severe inflammatory spinal cord lesions, whereas the brain remains relatively spared, which is very similar to NMOSD/MOGAD disease in humans. A gene expression profiling study sought to discern whether spontaneous OSE or MOG-induced EAE reproduced the genetic contribution to MS pathogenesis more closely, and concluded that the OSE model is probably linked more closely to human MS risk genes due to differentially higher expressed Th1 genes (281). A thorough gene expression profile for MOGAD still needs to be generated; however, the cytokine profile (see above) is rather indicative of a predominant Th17 response in MOGAD, which needs to be verified.

It has been suggested that most axonal damage in MOGAD happens during the initial attack, measuring neuroinflammatory biomarkers (such as MBP, sNFL, GFAP, and Tau), and relapses are associated with increased myelin damage (282). It has been suggested that antineurofascin antibodies contribute to axonal pathology in a passive transfer MOG-EAE model (283). It has been shown in double-transgenic OSE mice that when MOG is knocked out, the autoimmune response of MOG TCR-specific T cells is redirected toward the medium-sized neurofilament (NF-M) (278). Subsequently, the same group was able to demonstrate that due to inefficient exposure to two self-antigens, these bi-specific T cells managed to escape tolerization (284). Interestingly, there are only few reports of MOG-IgG/AQP4-IgG double positivity in MOGAD/NMOSD patients (285–287), and peripheral involvement in MOGAD is rarely reported (288).

The major drawback of TCR transgenic 2D2 mice and double transgenic OSE mice is that there is no complement deposition or granulocyte recruitment present (277, 279). Several humanized models have been established (265). It was shown in a transgenic mouse line that was generated to express human fragment crystallizable gamma receptors (hFcgRs) that recognize Immunoglobulin G antibodies, nicely reviewed in (289), that FcgRs but not complement activation contribute to EAE and that the exacerbation is dependent on MOG recognition by the human-derived antibodies (290). However, it is currently unclear which disease should be mimicked with this model, as it was shown that MS does not harbor anti-MOG autoantibodies and MOGAD probably has a complement-activating component driving lesion formation (13, 35) although the extent of complement involvement in human pathology is under debate.

Other transgenic mouse models investigated the relevance of IL-6, TH17 cells, oligodendrocytes, Nrf2, and CXCR3 (225, 227, 240, 291–293). The presence of MOG-IgG in MOGAD patients suggests B cell involvement that could be mirrored in several EAE models (294–297). SJL/J mice expressing a MOG_92−106_-specific transgenic TCR^1640^ with high frequency (99% proportion of transgenic Vα8.3^+^/Vβ4^+^CD4^+^ T cells) spontaneously produced pathogenic MOG-specific IgG1 antibodies (162).

### 4.7 MOG induced EAE in non-human primates

Different EAE models in monkeys have been reviewed elsewhere (298–300). EAE models developed in the rhesus macaque (*Macaca mulatta*) and the cynomolgus monkey (*Macaca fascicularis*) tend to replicate acute disseminated (leuko)encephalomyelitis well (301). In all non-human primates (NHP) the disease course varies with a more acute/relapsing or chronic disease course depending on the adjuvant used, complete or incomplete Freund's Adjuvants, respectively.

In the common marmoset monkey (*Callithrix jacchus*), extensive cortical demyelination could be induced upon immunization with rMOG_1−125_ and CFA (302). Lesions were dominated by macrophage/microglia activation and T cell infiltration (mostly perivascular) with few B cells, the cellular infiltrate was generally lower than in the parenchyma. Furthermore, IgG infiltration and complement deposition were observed. No subpial demyelination could be observed which is in contrast to patients with MOGAD, as well as in another study that observed subpial lesions in all experimental animals (303). Another study with marmoset monkeys immunized with rMOG_1−125_ and CFA found inflammatory lesions in cerebral white matter with some animals being affected also in the spinal cord and optic nerve. Lesion composition was similar with activated macrophage/microglia, T cell infiltrate, few B cells, IgG and complement deposition, and large confluent demyelinating lesions with some perivascular preference. The authors mentioned some axonal damage and indications for early remyelination (304). The encephalitogenic epitope inducing EAE in marmosets in mixed human myelin and CFA-induced immunization was shown to be MOG_14−36_ and not MBP (305); however, it could be shown that EAE could also be induced with myelin (from both WT and MOG^−/−^ C57BL/6 mice) but severity/disease progression were dependent on the presence of MOG-IgG (306). IL17-A production was found to be elevated compared to IFN-γ when marmoset monkeys were challenged with synthetic MOG_34−56_ peptide alone (307), which is in line with the cytokine profile suggested in MOGAD; however, although treatment with an anti-IL17-A antibody delayed onset of EAE, it did not abrogate its development (308). Another study found elevated levels of IL-6, G-CSF, IL-8, and IFN- γ in cynomolgus macaques immunized with rhMOG and IFA which was similar to CSF analyzed from children with acquired autoimmune disease positive for anti-MOG antibodies who had elevated levels of IL-6 and G-CSF (309).

### 4.8 Infection-induced animal models—Are they relevant models for MOGAD?

MOGAD has been associated with preceding infection or vaccination (310, 311) in ~20% of cases although a causal relationship to any specific agent has not been discerned yet. Recently, cases of MOGAD after infection or vaccination with COVID-19 vaccines (both mRNA and vector-based) were reported, some with detectable persistent long-term MOG-IgG (311–323). Different types of coronaviruses have been used extensively to induce EAE, resembling different aspects of MS/MOGAD in different species over the last six to seven decades to just give a few examples (205, 324–329). Biphasic disease with a short fulminant acute phase and a 1-month long chronic phase characterized by ongoing inflammatory demyelination can develop in mice infected with Theiler's murine encephalomyelitis virus (TMEV), which is not the case in all species (330–332). Similar to MOGAD, TMEV infection in mice features perivascular immune cell infiltrates, leptomeningeal and white matter mononuclear cell infiltrates in the spinal cord, and primary demyelination around day 15 after viral intracerebral inoculation (333–336). Spontaneously occurring ADEM-like disease could be observed in a Japanese macaque (JM) colony at the Oregon National Primate Research Center (ONPRC) that has been linked to infection by a gamma-herpesvirus, JM rhadinovirus (JMRV) (337). A case report from Japan with high titer MOG-IgG links influenza-A infection to longitudinally extensive TM (338).

Besides *M. tuberculosis* (339) and Pertussis toxin (induces IL-6 and reduces Treg compartment) (340) that are usually used for immune stimulation to induce EAE in mice, other infectious agents have been used prior or post-immunization with MOG_33−35_ like SEB (341) or LPS (342), exacerbation of MOG-induced EAE by intraperitoneal injections of a viral mimetic, polyinosinic-polycytidylic acid (PIC) (343), Cytomegalovirus infection (344) which induces susceptibility to EAE in resistant BALB/c mice (345), Influenza virus infection (346) by enhanced type I T cell infiltration. 2′-5′ oligoadenylate synthetase-like 1 (OASL1) deficient (Oasl1^−/−^) mice are resistant to viral infections, as OASL1 specifically inhibits the translation of interferon regulatory factor 7 (IRF7), the master transcription factor for interferon-1 (IFN-I). Thus, IFN-I production is negatively regulated upon viral infection and (Oasl1^−/−^) mice seem to have an enhanced resistance toward MOG-induced EAE (347). Protective effects toward EAE were also shown in a model of sepsis (348) and some malaria strains (349). Interestingly, it could be seen in a study by Nourbakhsh et al. that predominantly children seronegative for EBV presented with MOG-IgG (44%) compared to only 5.5% MOG+ in EBV+ children (350); likewise, no correlation between MOG+/EBNA+ was found in children in another study (351), suggesting that if infectious agents were involved/associated in the development of both diseases, they would be distinct. Cases linking LETM to *M. tuberculosis* infection have been reported (352, 353). Molecular mimicry between MOG_18−32_ and Semliki Forest Virus (SFV) could be demonstrated after demyelination-inducing immunization of C57Bl6/J mice (354). Infection with *S. pneumoniae* was shown to upregulate IL-6 and TNF-α in mice immunized with MOG_35−55_ (355).

In several animal models [Brown Norway rats challenged with MOG (356), rats challenged with replication-deficient adenovirus vector carrying IL-1β cDNA (AdIL-1β) (357)] a beneficial effect on EAE outcome was demonstrated with IFN beta-1a. It was also demonstrated in a mouse model (TMEV-infected SJL/J mice) that a shorter duration of treatment was associated with remyelination, whereas long-term treatment seemingly promoted demyelination (358).

## 5 Preclinical imaging in MOGAD animal models

Many clinical imaging methods can be applied to preclinical research in animal models with minimal adaptations. Additional methods beyond what is possible in clinical research allow imaging with higher, up to single-cell, resolution and better labeling of key players in pathophysiological processes. There are several applications for preclinical imaging: Firstly, comparing imaging features between MOGAD and its potential animal models can be used to validate the model's suitability. Secondly, imaging can be useful in traditional animal research investigating disease cause and pathophysiology by allowing longitudinal high-resolution analyses and the definition of time points based on imaging features, thereby reducing the number of needed animals. Thirdly, it can aid image marker development: New imaging methods can be tested in animal models for potential clinical application, especially regarding their safety, sensitivity, and correlation with histological features. When clinically established imaging methods are used to describe new distinct features in a disease, assumptions are often made about their pathophysiological origin. By back-translating these imaging methods and findings into an animal model, these assumptions can be tested using histology or molecular analyses. Finally, during drug development and testing, translatable methods can be extremely useful since future clinical trial endpoints can already be tested early on.

### 5.1 Brain and brainstem

As described above, actively induced MOG_35−55_-EAE (induced by MOG_35−55_, CFA, and PT) only has a low affection of the brainstem and cerebellum and mostly absent inflammation and tissue damage in the forebrain. Although this picture closely resembles the brain involvement of many MOGAD patients, it limits the use of this model for the investigation of MOGAD brain lesions. Using T1-weighted imaging with contrast enhancement, the brain involvement in 2D2^+^ mice was also shown to be little or non-existent (359). Only actively induced MOG_35−55_-EAE (induced by MOG_35−55_, CFA, and PT) in non-obese diabetic (NOD) mice, a model with relapsing-remitting disease course, leads to MRI gadolinium-enhanced lesions in T1-weighted imaging, located in corpus callosum, fimbria, and internal capsule (268). Although promising, this lesion pattern is more in line with MS pathology. In common marmoset monkeys, MOG_1−125_-induced EAE causes small T2 hyperintensities within the white matter with histopathologically confirmed demyelination, which can subsequently develop into expanding confluent lesions. This model might be suitable to model MOG-IgG seropositive ADEM, but further confirmatory research is warranted (268, 360).

Absent microstructural brain damage in actively induced MOG_35−55_-EAE in C57BL/6 mice (induced by MOG_35−55_, CFA, and PT) was confirmed by a DTI study, which did not detect differences in DTI parameters of anterior commissure, corpus callosum, cerebral peduncle, and external capsule between MOG_35−55_-EAE and controls (361). Similarly, the application of magnetization transfer ratio (MTR), which is suggested to be a sensitive method to detect demyelination, did not find any changes in actively induced MOG_1−125_-EAE in C57BL/6 mice (induced by MOG_1−125_, CFA, and PT) in line with absent histopathological findings, which is in contrast to results in monkeys described above (362). No in-depth diffusion-weighted MR studies in people with MOGAD exist yet. Lesion load, volumetric analyses, and diffusion-weighted imaging have also been applied in the preclinical testing of new and established therapeutic agents (363–366). Although easily translatable into clinical research, one has to be aware that preclinical MRI markers are not well-validated in distinct models so far.

However, some clinical imaging features of MOGAD patients can be reproduced: using serial post-contrast FLAIR (*fluid-attenuated inversion recovery)* sequences after gadolinium administration in actively induced MOG_35−55_-EAE (induced by MOG_35−55_, CFA, and PT) in C57BL/6 mice, Pol and colleagues showed leptomeningeal contrast enhancement in all mice that decreased during the chronic stage and correlated with the leptomeningeal invasion of macrophages as well as T- and B-cells in histology, which elucidates the leptomeningeal enhancement described in many MOGAD patients (367). Furthermore, two studies investigated the use of superparamagnetic iron oxide-enhanced MRI in MOG-EAE rats, which were actively induced by recombinant human MOG in 1AV1 congenic Lewis rats, and showed a demarcation of lesions in the cerebellum, brainstem, and periventricular regions, which were corresponding to lesional iron-laden macrophages in histology, suggesting that superparamagnetic iron oxide-enhanced MRI might be useful for the detection and demarcation of inflammatory CNS lesions (368, 369).

In the process of developing new imaging methods, preclinical research can help to establish the pathophysiological grounds. Especially when developing methods with potential side effects for patients, such as testing new positron emission tomography (PET) tracers, prior extensive preclinical research is warranted. In the CNS, translocator protein (TSPO) is thought to be mainly expressed in activated microglia cells, and TSPO ligands have been used to detect inflammatory CNS processes. Widespread accumulation of two different TSPO ligands was shown in actively induced MOG_35−55_-EAE in C57BL/6 mice (induced by MOG_35−55_, CFA, and PT) with and without additional cuprizone treatment including the spinal cord, cerebellum, cortex, striatum, and hippocampus (370, 371). Neuropathological analyses confirmed microglial activation and were correlated with tracer uptake, thereby validating the method. In a similar approach, tracers for CD19 and the cystine/glutamate antiporter were validated in actively induced MOG-based animal models in C57BL/6 mice (Hoehne et al.: MOG_35−55_, CFA, PT, Stevens et al.: MOG_1−125_, CFA, PT) (372, 373). Fluorinated molecules might be another promising and non-toxic option for MR-detectable tracers to study neuroinflammation in the near future (374–378).

Going one step further, preclinical research allows more invasive imaging approaches with up to single-cell resolution such as real-time confocal imaging and two-photon excitation microscopy. The latter uses the simultaneous non-linear excitation by two photons of fluorophores to report on the sequential order and interaction of different key players during a pathological process. Particularly interesting is the application in adoptive transfer models using autofluorescent lymphocytes, which can then be tracked longitudinally throughout the disease. By transferring MOG-sensitized lymphocytes isolated from green fluorescent protein (GFP)-transgenic mice to C57BL/6 mice, Yura et al. were able to track widespread invasion of these GFP-labeled CD4+ in the brain and spinal cord using confocal imaging and detected nearly exclusive production of T helper cell type 1 using real-time PCR (379). In a different approach, Siffrin and colleagues used the actively induced MOG_35−55_-EAE model (induced by MOG_35−55_, CFA, and PT) in mice with enhanced GFP (eGFP) expression in neurons and neuronal processes and red fluorescent protein in bone marrow-derived peripheral immune cells, as well as adoptive transfer models (stimulation performed with MOG_35−55_), to investigate neuron-immune cell interaction and to show that Th17 cells induce early neuronal damage (380).

### 5.2 Spinal cord

So far, only a few studies implemented preclinical spinal cord MRI: T1-weighted imaging with contrast enhancement was used to characterize spinal cord involvement in 2D2^+^ mice showing enhancement in half of the mice that correlated with histologically confirmed immune cell infiltration (359). Employing *in vivo* lumbar DTI, axial and radial diffusivity changes in line with microstructural axonal and myelin pathology in the spinal cord, respectively, have been shown in actively induced MOG_35−55_-EAE in C57BL/6 mice (induced by MOG_35−55_, CFA, only) and in an adoptive transfer model of MOG-reactive TH1 cells in C57BL/6 mice (stimulated with MOG_35−55_) (381, 382). In both models, exploratory treatments were suggested to improve DTI parameters toward control values, pointing toward a relative sensitivity of these metrics.

Spinal cord MRI has also been performed in two studies *ex vivo* post-fixation, potentially limiting morphometric analyses (383). Derdelinckx and colleagues treated actively induced MOG_35−55_-EAE in C57BL/6 mice (induced by MOG_35−55_, CFA, and PT) with myelin antigen-presenting tolerogenic dendritic cells and observed a stabilized EAE disability score and an inhibited T cell response (32). In this study, *ex vivo* gadolinium-enhanced spinal cord MRI was implemented post-fixation to confirm a reduced lesion load after treatment and to localize lesional and non-lesional tissue for histological analyses (32). Cahill and colleagues developed a new PPARα^mut/WT^ 2D2^+^ animal model with a mild relapsing-remitting disease course and increasing hind limb clasping during the disease process (384). Apart from histological analyses showing T cell and microglial activation as well as axonal and myelin damage at several locations in the brain, brainstem, spinal cord, and optic nerve, they also applied *ex vivo* post-fixation MRI analyses after 9 months to confirm spinal cord atrophy compared with 2D2^−^ littermates (384). Neither study generated imaging data that can easily be transferred/translated into clinical application.

In one recent study using advanced preclinical imaging, two-photon excitation microscopy was applied to the spinal cord in actively induced MOG_35−55_-EAE (induced by MOG_35−55_-EAE, CFA and PT) for the first time: Steudler et al. used *ODCmitoGFP-Tomato* mice, which have GFP-labeled mitochondria in tdTomato-labeled oligodendrocytes (385). They applied two-photon excitation microscopy to reveal the complex evolution of the mitochondrial redox state with increased and decreased oxidation at the preclinical and chronic stages, respectively, suggesting an early involvement of oligodendrocyte mitochondria in the inflammatory process in EAE (385).

### 5.3 Retina and optic nerve

Many techniques investigating the visual system in patients can directly be translated to their application in animals with only minimal technical adaptations, for example, to correct for differences in refraction. When back-translating OCT imaging to rodents, the inner retinal layer (IRL) is usually quantified, instead of separating pRNFL and GCIPL, due to the lower retinal neuroaxonal content and lower resolution in mice. Cruz-Herranz et al. performed comparative OCT in different neuroinflammatory mouse models: Actively induced MOG_35−55_-EAE (induced by MOG_35−55_, CFA, and PT) in C57BL/6 mice led to severe thickening of the IRL with subsequent thinning; a 32% retinal ganglion cell loss within 120 days (54% in 9 months) and T cell and microglia invasion were later confirmed by histopathology (386). In contrast, actively induced MBP-EAE (induced by MBP, CFA, and PT) led to a much milder disease course with stable IRL measurements and no retinal ganglion cell loss. Active MOG_35−55_ -EAE induction in TCR^2D2^ mice (induced by MOG_35−55_, CFA, and PT) led to an earlier IRL thinning without edema, yet the extent (49% within 120 days) was nearly comparable with C57BL/6 mice after MOG_35−55_ -EAE induction (386). Uninduced TCR^2D2^ mice also underwent IRL thinning and thereby neurodegeneration within a 120-day period suggesting an underlying process in the mouse line (386). In a similar fashion, actively induced PLP_139−151_-EAE in SJL/J mice (induced by PLP_139−151_, CFA, and PT) led to IRL atrophy, yet wild-type uninduced SJL/J mice also showed IRL thinning (386). This is most likely due to a homozygous Pde6b^rd1^ mutation for retinopathy these mice carry (386). In marmoset monkeys actively induced with MOG_1−125_-EAE (induced by recombinant rat MOG_1−125_ and CFA), only 50% have an ON at all (387). Taken together, Cruz-Herranz and other independent studies imply a strong resemblance of actively induced MOG_35−55_-EAE in C57BL/6 with adult MOGAD-ON, describing features such as early edema and severe neuroaxonal loss over an extended period after ON, while other models might closer resemble the milder course in MS-ON (386).

Actively induced MOG-EAE models gained further stand as a MOGAD model by a recent study confirming bilateral ON in 70% of MOG_1−125_-EAE in Brown Norway (BN) rats (induced by MOG_1−125_, CFA only) using visually evoked potentials (VEPs) (388). In Dark Agouti rats actively induced with MOG_1−125_-EAE (induced by MOG_1−125_, CFA only), a VEP latency delay could be observed even before first motor deficits were present, i.e., during an inflammatory state, demyelination and axonal loss were observed at later disease stages (389). Severe ON was caused in BN rats actively induced with the same model (390). Induced apoptosis of retinal ganglion cells (RGCs) in this model in BN rats could be seen as independent of optic nerve involvement (391). Two additional studies employing OCT and histopathology measured early, inflammation-preceding, RNFL thickness reduction in this actively induced MOG_1−125_-EAE model in BN rats (induced by MOG_1−125_, CFA only) (392, 393), respectively. Later, an increase in oligodendrocyte alphaB-crystallin, a heat-shock protein induced by cellular stress, was observed during the preclinical stages, particularly in the optic nerve head in this actively induced MOG_1−125_-EAE model in BN rats (induced by MOG_1−125_, CFA only) (394). This is in line with measurements gained in an MS study (395, 396). Contrary to these observations, RGC loss induced in C57/B6 mice by actively induced MOG_35−55_ –EAE (induced by MOG_35−55_, CFA, and PT) occurred only in late stages of the disease (post-immunization day 42), whereas CD4+Tcell infiltration, demyelination, microglial, and astrocyte activation were induced in the optic nerve by PID 16 (397). Further late events include degeneration of retinal neurites and synapses as well as glial cell activation in the inner retina. Similarly, in actively induced PLP_139−151_-EAE in SJL/J mice (induced by PLP_139−151_, CFA, and PT), RGC loss was detected by PID14, which in this model was however after cell infiltrates had been detected in the optic nerve around PID 9, pointing toward inflammation preceding RGC loss in this model (398).

As a potentially promising development for pediatric MOGAD-ON, the OSE model shows good results: OCT in OSE mice with spontaneous encephalomyelitis starting on day 26 after birth showed retinal neurodegeneration, which was confirmed by histopathology as 38% loss at 6 weeks of age (399). The functional relevance of RGC loss was confirmed by electroretinogram (ERG) (399).

Due to the close correlation between structural and functional metrics, multimodal assessment including OCT and functional assessments is common in rodents. Functional metrics back-translated from clinical applications include ERGs and VEPs, usually performed as flash-VEP. As a metric for vision in mice, the optomotor response (OMR) is assessed, which quantifies the compensatory head movement when the mouse is exposed to a moving light-dark pattern. Despite being the current gold standard for vision in mice, the OMR was critiqued for (A) the interference of vision and motor function, (B) the overlay with the optokinetic response, and (C) not depicting the (retina–lateral geniculate nucleus—primary visual cortex)-pathway usually associated with vision in humans. Outputs of VEP, ERG, and OMR have been shown to correlate very well with the neuroaxonal content measured by OCT and by histopathology, for example, in actively induced MOG_35−55_-EAE in mice (induced with MOG_35−55_, CFA, and PT) (400, 401).

Applying visual outcome parameters in animal research currently serves two major purposes: Firstly, we can use animal models to better understand the pathophysiological basis of our functional metrics. Recently, VEP became an outcome parameter for myelin in clinical trials investigating potentially remyelinating agents. Although the measurement of conduction speed seems like a feasible metric for myelin, the pathophysiological basis of this assumption was never validated and the sensitivity of VEPs for myelin content was never shown. Using different demyelinating animal models including actively induced MOG_35−55_-EAE in C57BL/6J mice (induced with MOG_35−55_, CFA, PT), Cordano and colleagues now demonstrated that quantitative measurements of myelination and remyelination correspond well with VEP latency, thereby validating it as a tool (402). This VEP change also correlates well with the dysregulation of potassium channels around the nodes of Ranvier as shown during inflammatory demyelination in actively induced MOG_35 − 55−_EAE (induced by MOG_35−55_, CFA, and PT) (403, 404). So far, only one VEP study has been performed in actively induced MOG_1−125_-EAE in marmoset monkeys (induced by rat recombinant MOG_1−125_, CFA only). Unfortunately, this study only reports a loss of amplitudes in line with neurodegeneration in the later course of the disease but does not report potential latency delays (405).

Secondly, functional outcome parameters can be used in animal research to show functional relevance very early in the development and testing of new therapeutic agents. The visual system is especially suitable for early drug testing for neuroprotective agents due to the clear association of one localized lesion in the optic nerve with subsequent neurodegeneration in the retina and functional decline (406–411). A single study also used the rodent visual system in actively induced MOG_35−55_-EAE transgenic mice backcrossed to a C57BL/6 background (induced with MOG_35−55_, CFA, and PT) to investigate the functional effects of remyelinating with the agent chloroindazole using VEP and ERG, yet the structure-function correlation was less robust (412). The only study so far using the rodent visual system in actively induced MOG_35−55_-EAE in mice (induced with MOG_35−55_, CFA, PT) to investigate the effects of anti-inflammatory treatment with anti-IL-17 antibodies showed that retinal neurodegeneration as measured by OCT, but not motor symptoms, was completely prevented by neutralizing IL-17 (413). This is particularly interesting since MOGAD patients were shown to have more IL-17-positive central memory cells than healthy controls with a particular increase in IL-17-positive IFN- γ positive central memory cells during relapses, again suggesting important parallels between MOG-EAE and MOGAD (282).

Optic nerve MRI using T1- and T2-weighted imaging has been validated in actively induced MOG_35−55_-EAE in C57Bl/6 mice (induced with MOG_35−55_, CFA, and PT) but sparsely performed (414). Qi et al. were able to establish volumetric optic nerve analysis using T1-weighted 3D 4.7-tesla MRI (415). As suggested from clinical experience, they were able to show significant optic nerve swelling and subsequent volume loss in an EAE model induced by CFA and homologous spinal cord emulsion. Reducing mitochondrial reactive oxygen stress by increasing SOD2 gene expression using virally mediated gene transfer led to less edema and prevented significant volume loss, which the analysis was sensitive enough to detect (415). The involvement of the optic nerve, optic tract, and chiasm was also shown for 2D2^+^ mice by contrast-enhanced T1-weighted imaging.

Interestingly, DTI has been applied to the visual system in rodents but not yet specifically in MOGAD patients. Manogaran et al. performed a multimodal study including OCT, T2-weighted imaging, and DTI in actively induced MOG_35 − 55−_EAE in C57BL/6J mice (induced by MOG_35−55_, CFA, and PT). They confirmed signal increase around the optic nerve in T2-weighted MRI in line with significant inflammation. DTI showed a decrease in axial diffusivity and an increase in radial diffusivity in the optic nerve and optic tract compared with controls. These changes were correlated with neuroaxonal parameters from OCT (416). DTI changes were confirmed by other independent studies in actively induced MOG_35 − 55−_EAE (induced by MOG_35−55_, CFA, and PT) (361, 417). A newer diffusion MRI approach called diffusion basis spectrum imaging (DBSI) was specifically developed to separate axonal and inflammatory pathologies. In its first application in actively induced MOG-EAE in C57BL/6J mice (induced by unspecified MOG peptide, CFA, and PT), the DBSI data suggest that axonal loss in ON occurs early and in parallel to the optic nerve edema (417). The application of DTI to the visual system in people with MOGAD is still pending.

The possibilities of retinal imaging in rodents exceed the options in clinical research. One example is confocal scanning laser ophthalmoscopy (CSLO), which is a non-invasive technique for real-time imaging of autofluorescent targets in the retina. In actively induced MOG_35 − 55−_EAE (induced by MOG_35−55_, CFA, and PT), CSLO has been applied to track myeloid cells in CX3CR1^GFP/−^ mice (expressing a green fluorescent protein under control of the endogenous CX3C locus chemokine receptor 1) (418, 419). CSLO was then used to characterize microglial activation longitudinally during the course of actively induced MOG_35 − 55−_EAE and to define time points of maximum microglial activation for further analyses (418). In the long term, this imaging method might be used with different targets and animal models. The more invasive alternative with better single-cell tracking is two-photon excitation microscopy, which can nowadays also be co-registered with OCT (420). Yet, it has been so far only applied to actively induced experimental autoimmune uveitis in CX3CR1^eGFP/−^ mice [induced by IRBP_1−20_ (*interphotoreceptor retinoid-binding protein*), CFA, and PT], an inflammation localized in the iris and ciliary body (421). Uveitis also occurs in MOGAD patients (422) and MOGAD-depicting animal models (292). Histopathological findings in uveitis are comparable in actively induced MOG_35−55_-EAE in (C57BL/6 x SJL) F1 and C57BL/6 mice (induced by MOG_35−55_, CFA, and PT) and mice with passive transfer of T cells specific to MOG_35−55_, suggesting a T-cell-mediated origin of autoimmune uveitis in MOGAD (423). Translational imaging including co-registered OCT and two-photon excitation microscopy can help to further elucidate the cause.

## 6 Concluding remarks

Separating MOGAD as a disease entity presents a unique challenge since researchers have investigated MOG-IgG-based animal models and MOG-IgG seropositive patients for decades as models for or as part of other conditions. This review is a first step toward understanding how the generated knowledge is specifically applicable to MOGAD. Translational imaging in MOGAD has provided useful information on disease pathophysiology, commonalities between animal models and disease, and potential imaging markers. Yet, true translational imaging research including clinical and preclinical aspects within the same study is still warranted. Also, many open questions remain such as: (1) Is the histopathology of the optic nerve and spinal cord comparable between MOG animal models and MOGAD patients (due to the lack of human pathology studies), and which would be the closest to reflect human disease? (2) What causes the gray matter involvement in MOGAD? (3) Is there a relevant portion of MOGAD patients developing a clinically progressive disease course and do we need a disease-specific definition of neuropathological progression? and (4) Should current treatment regimens for MOGAD be reevaluated because (A) no adverse events to, e.g., Fingolimod/Natalizumab (as seen in AQP4-IgG seropositive NMOSD) were observed in MOG-IgG seropositive patients (217) and (B) many treatments have been shown to be beneficial in MOG-induced EAE that are less used in or have been unsuccessful in MS (160, 423–425). In the future, translational and advanced imaging might provide answers to these questions and support the development of biomarkers for the diagnosis and monitoring of MOGAD.

## Author contributions

FO and MH participated in the original conceptualization and initial draft of the manuscript. FP contributed to substantial revisions of the manuscript. All authors contributed to the revisions of the manuscript and approved the submitted version.
