# Erratum: Delimiting MOGAD as a disease entity using translational imaging

**DOI:** 10.3389/fneur.2024.1390688

**Published:** 2024-03-07

**Authors:** Frontiers Production Office

**Affiliations:** Frontiers Media SA, Lausanne, Switzerland

**Keywords:** myelin oligodendrocyte glycoprotein associated disease, imaging, translational research, EAE, animal models

Due to a production error, minor text corrections were implemented throughout the article.

A correction has been made to the section “Concluding remarks,” where the question numbers were inadvertently linked to the reference numbers. The section is reproduced below:

“Also, many open questions remain such as: (1) Is the histopathology of the optic nerve and spinal cord comparable between MOG animal models and MOGAD patients (due to the lack of human pathology studies), and which would be the closest to reflect human disease? (2) What causes the gray matter involvement in MOGAD? (3) Is there a relevant portion of MOGAD patients developing a clinically progressive disease course and do we need a disease-specific definition of neuropathological progression? and (4) Should current treatment regimens for MOGAD be reevaluated because (A) no adverse events to, e.g., Fingolimod/Natalizumab (as seen in AQP4-IgG seropositive NMOSD) were observed in MOG-IgG seropositive patients (217) and (B) many treatments have been shown to be beneficial in MOG-induced EAE that are less used in or have been unsuccessful in MS (160, 423–425).”

Due to a production error, the references for “(191, 406) and (408)” were incorrectly written as “Lopez-Chiriboga AS, Majed M, Fryer J, Dubey D, McKeon A, Flanagan EP, et al. Association of MOG-IgG serostatus with relapse after acute disseminated encephalomyelitis and proposed diagnostic criteria for MOG-IgG-associated disorders. JAMA Neurol. (2018) 75:1355–63. doi: 10.1001/jamaneurol.2018.1814”, “Talla V, Koilkonda R, Guy J. Gene therapy with single-subunit yeast NADH-ubiquinone oxidoreductase (NDI1) improves the visual function in experimental autoimmune encephalomyelitis (EAE) mice model of multiple sclerosis (MS). Mol Neurobiol. (2020) 57:1952–65. doi: 10.1007/s12035-019-01857-6,” and “Talla V, Koilkonda R. Targeted Kruppel-like factor 4 gene knockout in retinal ganglion cells improves visual function in multiple sclerosis mouse model. eNeuro. (2020) 7:ENEURO.0320-19.2020. doi: 10.1523/ENEURO.0320-19.2020”. These should be “Wendel EM, Thonke HS, Bertolini A, Baumann M, Blaschek A, Merkenschlager A, et al. Temporal dynamics of MOG antibodies in children with acquired demyelinating syndrome. *Neurol-Neuroimmunol*. (2022) 9:e200035. doi: 10.1212/NXI.0000000000200035”, “Talla V, Porciatti V, Chiodo V, Boye SL, Hauswirth WW, Guy J. Gene therapy with mitochondrial heat shock protein 70 suppresses visual loss and optic atrophy in experimental autoimmune encephalomyelitis. *Invest Ophthalmol Vis Sci*. (2014) 55:5214–26. doi: 10.1167/iovs.14-14688” and “Talla V, Koilkonda R. Targeted Krüppel-like factor 4 gene knock-out in retinal ganglion cells improves visual function in multiple sclerosis mouse model. *eNeuro*. (2020) 7:ENEURO.0320-19.2020. doi: 10.1523/ENEURO.0320-19.2020”. Reference 425 and reference 292 were the same and therefore reference 425 was deleted. A total of 11 references (76, 78, 82, 86, 97, 107, 113, 126, 128, 130 and 132) indicated the first published date and not the online issue date.

The publisher apologizes for this mistake. The original article has been updated.
